# Pediatric Collagenous Gastroenterocolitis Successfully Treated with Methotrexate

**DOI:** 10.1155/2020/1929581

**Published:** 2020-02-22

**Authors:** Beate C. Beinvogl, Jeffrey D. Goldsmith, Ramalingam Arumugam, Michelle Kennedy, Mani Mokalla, Paul A. Rufo, Menno Verhave

**Affiliations:** ^1^Division of Gastroenterology, Boston Children's Hospital, Boston, MA, USA; ^2^Department of Pathology, Boston Children's Hospital, Boston, MA, USA; ^3^Minnesota Gastroenterology, PA, St. Paul, MN, USA; ^4^Children's Hospitals and Clinics of Minnesota, Minneapolis, MN, USA

## Abstract

A two-and-one-half-year-old previously healthy female presented with a ten-week history of watery diarrhea, nonbilious and nonbloody emesis, and low-grade fevers. She was found to have severe hypoalbuminemia and hypogammaglobulinemia. Her symptoms persisted, and she became dependent on parenteral nutrition. Biopsies obtained during subsequent endoscopic and colonoscopic studies revealed findings consistent with collagenous gastroenterocolitis. She responded to an empiric course of prednisone, but her symptoms recurred shortly after transitioning to oral budesonide. After successful reinduction with intravenous prednisone, intramuscular methotrexate was initiated. She remained asymptomatic during a 15-month course of therapy, and she continued to do well clinically until approximately nine months after weaning off methotrexate. At that point, she experienced a recurrence of diarrhea, and repeat endoscopic evaluation confirmed collagenous colitis. This responded nicely to a short course of oral budesonide, and she has since remained asymptomatic and off any therapy.

## 1. Introduction

Collagenous inflammatory conditions are rare disorders characterized by a finding of increased subepithelial collagen deposition, typically with an associated inflammatory infiltrate, in the lamina propria of the stomach and intestines [1]. Most pediatric cases in the literature have reported collagenous disease limited to the mucosa of the stomach with a generally benign prognosis. Involvement of the entire gastrointestinal tract, as described in this case report, is exceedingly rare and can be life-threatening. The few case reports that have been published concerning diffuse collagenous gastrointestinal disease in children have all described severely ill young patients, all of whom required systemic immunosuppression and displayed inconsistent clinical response. [2–4] We present here the case of a 2.5-year-old female with steroid-refractory collagenous gastroenterocolitis who was successfully treated with intramuscular methotrexate. Informed consent was obtained from the family for publication.

## 2. Case Presentation

A 2.5-year-old previously healthy female presented with a ten-week history of passing ten watery stools per day, one week of nonbilious and nonbloody emesis, and low-grade fevers. Her vital signs were normal. However, her physical exam was notable for decreased skin turgor, a distended but nontender abdomen, and normal bowel sounds. She had a history of mild constipation, two prior ear infections, and one episode of pneumonia requiring oral antibiotic therapies.

Her laboratory studies were significant for hypoalbuminemia (albumin 1.5 g/dL (3–4.6)) and hypogammaglobulinemia (IgA < 7.3 mg/dL (20–230), IgE 23.4 units/mL (0–30), IgG 203 mg/dL (400–1300), and IgM < 3.6 mg/dL (30–120)). A complete blood count showed lymphopenia but was otherwise normal. Lymphocyte subsets were notable for borderline low CD3^+^ and CD3^+^/CD4^+^ T-cell subsets and elevated CD5^+^/CD19^+^ B-cell subsets. She had irregularities of her T- and B-cell memory panels, and she was noted to have nonprotective titers to varicella although she had been immunized about a year prior. The regulatory T-cell panel was normal. Her C-reactive protein was elevated (CRP 12.2 mg/dL (0–0.3)). A basic metabolic profile, hepatic panel, and serum lipase were otherwise unremarkable. Celiac serologies were uninterpretable in the context of her hypogammaglobulinemia. Specific IgE testing for milk, soy, shellfish, egg, nuts, corn, and wheat were all negative. Her stool tested negative for occult blood, and her fecal elastase and alpha-1 antitrypsin levels were in the normal range. Infectious stool studies including *Clostridium difficile*, routine pathogens, giardia, and cryptosporidia stains, as well as ova and parasite analyses, were all negative. Urinalysis revealed no proteinuria. An abdominal ultrasound was notable only for numerous fluid-filled bowel loops. The gross endoscopic appearance of her esophagus, stomach, and small and large intestine was unremarkable. However, mucosal biopsies revealed marked duodenal villous atrophy and crypt elongation in the absence of intraepithelial lymphocytes. Colonic biopsies demonstrated increased lamina propria eosinophils in the sigmoid and descending colon in the absence of thickened subepithelial collagen. Disaccharidase analysis showed deficiency of all enzymes, consistent with the villous blunting.

She was treated empirically with a five-day course of systemic steroids to address presumed celiac crisis. She was also placed on a gluten and dairy-free diet, and the majority of her nutrition was derived from elemental formula delivered via a nasogastric (NG) tube. Supportive care also included initiation of cyproheptadine to assist with gastric accommodation as well as lansoprazole for any contributory peptic symptoms. However, she was unable to tolerate her enteral feeds, her hypoalbuminemia worsened, and she became progressively more edematous. As a result, placement of a central line and initiation of total parenteral nutrition (TPN) became necessary within one week of her admission.

**Figure 1:**
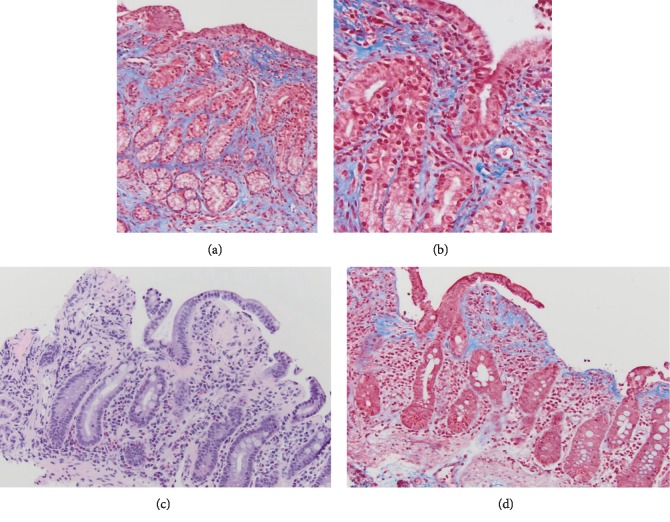
Histopathologic findings of collagenous gastroenterocolitis. Increased subepithelial collagen. (a) Collagenous gastritis (stomach, trichrome). (b) Collagenous gastritis, high magnification (stomach, trichrome). (c) Collagenous duodenitis (duodenum, H&E). (d) Collagenous duodenitis (duodenum, trichrome).

Repeat upper endoscopic and colonoscopic evaluations were performed five weeks after the initial studies, and mucosal biopsies obtained at this time revealed granularity in the mucosal lining of the stomach, as well as villous blunting in the terminal ileum. Markedly thickened subepithelial collagen, consistent with collagenous gastroenterocolitis as well as a mixed lamina propria inflammatory infiltrate, was noted in the mucosa of stomach, duodenum, terminal ileum and colon (Figure 1).

The patient was started on IV methylprednisolone (1 mg/kg, twice daily) for the next two weeks, during which she experienced a rapid resolution of symptoms and normalization of her serum albumin and immunoglobulin levels. Her overall gut function improved, her TPN was discontinued, and she was transitioned to oral prednisolone (1 mg/kg, twice daily) for two weeks and subsequently tapered off over the next four weeks. In conjunction, she was started on a maintenance regimen inclusive of an oral budesonide slurry, budesonide extended-release capsules (3 mg), and a gluten-free diet. Findings from follow-up upper endoscopic and colonoscopic evaluations performed two months after the diagnostic endocosopy revealed grossly and histologically normal-appearing mucosa.

Within two weeks of completing the steroid taper, however, she again developed diarrhea. She was rehospitalized and once again responded readily to IV corticosteroid therapy. Given the apparent steroid dependency of her disease, she was placed on weekly intramuscular doses of methotrexate (15 mg/m^2^) along with daily folate supplementation. She successfully weaned off steroids, and biopsies obtained during a repeat endoscopy performed six months later revealed normal mucosa. She experienced no medication-related side effects, and she manifested appropriate weight gain, linear growth, and physical development. After remaining in full clinical remission during a course of 15 months of weekly methotrexate therapy, this immunomodulator was discontinued. She remained well but experienced a mild flare of collagenous colitis about nine months later (endoscopically confirmed). She responded nicely to a two-month course of oral budesonide (6 mg/day). Oral budesonide was chosen due to mild symptoms and absence of any small bowel involvement. Dairy and gluten were successfully reintroduced, and she has remained in clinical remission for the past two years.

Follow-up laboratory testing demonstrated a resolution of her hypogammaglobulinemia, as well as an improvement of clinical symptoms attributed to protein losing enteropathy. There was a normalization of her lymphocyte subsets, and she demonstrated a satisfactory response to reimmunization with pneumococcal and tetanus vaccines. Reimmunization with live vaccines was deferred while on methotrexate. T-cell function improved with good proliferation to phyotohemagglutinin (PHA), but she remained anergic to pokeweed mitogen proliferation (PWM). Additional antigen testing is ongoing to clear her to receive repeat live vaccines. She has not had any other infections suggestive of immune deficiency.

## 3. Discussion

There are several case series in the literature describing children with isolated collagenous gastritis, with most patients experiencing a generally benign clinical course. [5–7] In contrast, collagenous mucosal disease involving the entire gastrointestinal tract, as described in this case report, is extremely rare and presents as a distinct clinical and prognostic phenotype. Only three other pediatric case reports exist in the literature to date (Table 1) [2–4]. All reported pediatric cases were young children between the ages of 9 months and 2.5 years and presented with similar clinical complaints of watery diarrhea, vomiting, weight loss, fevers, hypoalbuminemia, and peripheral edema. [2–4].

The pathogenesis of collagenous mucosal inflammatory diseases is poorly understood. Various hypotheses regarding the deposition of subepithelial collagen exist. Some investigators have suggested that the deposition occurs as a result of a reparative process in response to an earlier inflammatory, autoimmune, infectious, or toxic damage [1, 8]. Similar to other cases in the literature, our patient had normal or nonspecific endoscopic or biopsy findings early in the disease course. This suggests that histologic collagen deposition may be a delayed occurrence in the course of these diseases and possibly a nonspecific histologic endpoint resulting from an underlying chronic inflammatory process. An immune-mediated process of collagen deposition has been suggested given reported associations with other autoimmune conditions, including celiac disease and inflammatory bowel disease [7, 9, 10]. Pulimod et al. identified degranulating eosinophils and mast cells in their ultrastructural study of gastric mucosa in patients with collagenous gastritis, and they suggested that degranulation in response to an unidentified antigen could be driving tissue damage and fibroblast proliferation [11].

An association of collagenous gastroduodenitis with combined variable immunodeficiency disease (CVID) has been described in adults [12]. The patient described in this vignette initially presented with hypogammaglobulinemia, abnormal lymphocyte subsets, and nonprotective immunization titers, initially raising concern for immune dysregulation. However, other findings typical of patients presenting with immunodeficiency with gastrointestinal involvement were not noted. The hypogammaglobulinemia resolved with successful corticosteroid and immunomodulatory treatment, most likely a result of decreased losses as the protein losing enteropathy resolved. Follow-up immunology testing and the absence of any further infections make an underlying immune dysregulation unlikely.

Given the rarity of diffuse gastrointestinal collagenous disease and the poor understanding of its natural history, there are no existing treatment algorithms to guide clinical therapy. Treatments that have been used for collagenous mucosal disease include ranitidine, omeprazole, sucralfate, aminosalicylates, sulfasalazine, bismuth subsalycilate, steroids, iron supplementation, cholestyramine, hypoallergenic diets, gluten-free diet, and parenteral nutrition with unclear benefit [5, 7]. The frequent response to steroids, particularly in patients with severe and extensive disease, suggests an underlying inflammatory process [1]. Reports of favorable responses to anti-TNF therapy or thiopurines in cases of adults with severe collagenous duodenitis have been described, further supporting the potentially beneficial role of systemic immunosuppressive agents to treat this condition [13, 14].

All reported pediatric patients with extensive mucosal involvement required hospitalization, most required at least temporary parenteral nutrition support, and all responded to systemic corticosteroid therapy (Table 1). The follow-up periods reported ranged from 14 weeks to 14 years, and individual patients displayed variable clinical courses. Leiby et al. reported a two-year-old who failed to wean off systemic steroids but responded favorably to oral bismuth subsalicylate. However, the histologic outcome data for this patient were not reported [3]. Almadhoun et al. reported the case of a 15-month-old toddler who experienced full clinical resolution in response to treatment with systemic IV and oral corticosteroid therapy and subsequently transitioned successfully to oral budesonide therapy. However, this patient failed to wean from budesonide therapy, required reinduction with systemic corticosteroids, and remained on budesonide one year after diagnosis [4]. The most extended clinical follow-up in the literature was reported by Billiémaz et al. in their description of a case of a nine-month-old infant who experienced progressive disease despite chronic steroid treatment (alternating systemic steroids and budesonide). Follow-up endoscopic study demonstrated gastric atrophy and the persistence of subepithelial collagen deposition. The patient ultimately experienced a full clinical remission on a combination of parenteral nutrition and gut rest. However, there is no data indicating whether or not there was any parallel improvement in histology in response to therapy (Table 1) [2].

The patient described in the present case report was followed for a total of 4 years. She initially had a full clinical and histologic response to systemic steroid therapy. However, she failed maintenance therapy with oral budesonide, required reinduction with corticosteroids, and was then maintained successfully with oral methotrexate for 15 months before being weaned off immunosuppressives entirely. A subsequent mild flare of collagenous colitis responded to a brief course of budesonide.

Existing reports demonstrate that most patients with extensive collagenous gastrointestinal disease will require at least a temporary course of steroid-sparing immunosuppressive therapy. Methotrexate has proven useful in the management of adults with refractory collagenous colitis, and we now report the first case of a child with extensive collagenous mucosal involvement successfully treated with methotrexate [15]. Methotrexate was chosen due to existing data demonstrating its favorable risk/benefit profile for use in the treatment of children with inflammatory and rheumatologic diseases [16, 17].

More studies are needed to understand the natural history of patients presenting with extensive collagenous mucosal inflammatory disease. In particular, there is a need to develop more standardized approaches to acute and chronic immunosuppressive therapy, including the use of steroid-sparing agents such as methotrexate.

## Figures and Tables

**Table 1:** Cases of pediatric collagenous gastroenterocolitis reported in the literature.

Study	Age of onset/gender	Presentation	Treatment	Follow-up time	Outcome
	Disease extent				
Leiby et al. [3]	2 y/M Gastroduodenocolitis	Watery diarrhea (2 mo)	IV steroids for 2 weeks, followed by PPI, mesalamine, PO steroids for 8 wks (response, but failed wean), bismuth subsalicylate (response)	14 wk	*2 wk:* Clinical resolution, biopsies with less inflammation but increased collagen band persisted. Relapsed 2 weeks after discontinuing systemic steroids. Then bismuth subsalicylate for 4 weeks with resolution of symptoms.
		Emesis (1 mo)			
		Low-grade fevers (1 mo)			
		Hypoalbuminemia (3.1 g/dL)			
		Elevated ESR			

Billiémaz et al. [2]	9 mo/M Gastro-duodeno-ileocolitis	Watery diarrhea Intermittent emesis and diarrhea since age 2 weeks Poor weight gain Hypoalbuminemia (1.4 g/dL) Hypogammaglobulinemia Lymphocytosis	13 yrs of prednisone 0.5–1 mg/kg/d, alternating with budesonide 3-4 mg/d, overnight enteral nutrition, gluten-free diet (persistent symptoms), TPN (full clinical improvement)	14 y	*3 y:* Gastric pseudopolyps with inflammatory areas in the gastric body, areas of mucosal erythema in the colon, histopathology not available
					*10 y:* Thick small tubular stomach, diffuse atrophic (extending into duodenum), erythema and multiple small submucosal nodules. Colon pale, thickened and nodular with disappearance of normal vascular pattern. Histology with atrophic mucosa and increase in the subepithelial collagen layer to 12–100 *µ*m.
					*14 y*: Asymptomatic on TPN and off steroids within 2 mo.

Almadhoun et al. [4]	15 mo/F Gastro-duodeno-ileocolitis	Diarrhea (4 wk)	Budesonide 3 mg for 5 days (no response) TPN, gut rest IV methylprednisolone 2 mg/kg (response) PO prednisone 1 mg/kg/dose with 2 mo steroid taper and oral budesonide 3 mg (remained asymptomatic) Symptom recurrence after weaning of budesonide, requiring reinduction with oral steroid, maintained on oral prednisone and budesonide 3 mg	1 y	*5 mo* (on budesonide): Persistent collagenous colitis without gastric or duodenal involvement.
		Peripheral edema (2 wk)			*1 yr* (on budesonide): Normal biopsies
		Emesis (1 wk)			
		Hypoalbuminemia (2.4 g/dL)			
		Stool alpha-1 antitrypsin >1.33 mg/g [<0.62]			

Current case	2.5 y/F Gastro-duodeno-ileocolitis	Diarrhea (10 wk)	IV methylprednisolone for 2 weeks, followed by PO steroids (response) Symptom recurrence after tapering of PO steroids, unable to maintain on budesonide. Reinduction with IV steroids and maintenance on methotrexate (response)	4 y	*2 mo:* Asymptomatic and normal pathology (on steroids)
		Emesis (1 wk)			
		Low-grade fevers			
		Hypoalbuminemia (1.5 g/dL)			
		Hypogammaglobulinemia			
					*9 mo:* Asymptomatic and normal pathology (after 6 mo of MTX)
					*17 mo:* Recurrence of collagenous colitis, good response and symptom resolution on oral budesonide
					*4 y:* Asymptomatic off treatment

PPI: proton-pump inhibitor; IV: intravenous; PO: enteral; TPN: total parenteral nutrition.

## References

[1] Freeman H. J. (2005). Collagenous mucosal inflammatory diseases of the gastrointestinal tract. *Gastroenterology*.

[2] Billiémaz K., Robles-Medranda C., Le Gall C. (2009). A first report of collagenous gastritis, sprue, and colitis in a 9-month-old infant: 14 years of clinical, endoscopic, and histologic follow-up. *Endoscopy*.

[3] Leiby A., Khan S., Corao D. (2008). Clinical challenges and images in GI. Collagenous gastroduodenocolitis. *Gastroenterology*.

[4] Almadhoun O. F., Katzman P. J., Rossi T. (2014). Collagenous colitis associated with protein losing enteropathy in a toddler. *Case Reports in Gastrointestinal Medicine*.

[5] Kamimura K., Kobayashi M., Sato Y. (2015). Collagenous gastritis: review. *World Journal of Gastrointestinal Endoscopy*.

[6] Hijaz N. M., Septer S. S., Degaetano J. (2013). Clinical outcome of pediatric collagenous gastritis: case series and review of literature. *World Journal of Gastroenterology*.

[7] Matta J., Alex G., Cameron D. J. S., Chow C. W., Hardikar W., Heine R. G. (2018). Pediatric collagenous gastritis and colitis: a case series and review of the literature. *Journal of Pediatric Gastroenterology and Nutrition*.

[8] Rustagi T., Rai M., Scholes J. V. (2011). Collagenous gastroduodenitis. *Journal of Clinical Gastroenterology*.

[9] Chutkan R., Sternthal M., Janowitz H. D. (2000). A family with collagenous colitis, ulcerative colitis, and Crohn’s disease. *The American Journal of Gastroenterology*.

[10] Roubenoff R., Ratain J., Giardiello F. (1989). Collagenous colitis, enteropathic arthritis, and autoimmune diseases: results of a patient survey. *The Journal of Rheumatology*.

[11] Pulimood A. B., Ramakrishna B. S., Mathan M. M. (1999). Collagenous gastritis and collagenous colitis: a report with sequential histological and ultrastructural findings. *Gut*.

[12] Daniels J. A., Lederman H. M., Maitra A., Montgomery E. A. (2007). Gastrointestinal tract pathology in patients with common variable immunodeficiency (CVID). *The American Journal of Surgical Pathology*.

[13] Schmidt C., Kasim E., Schlake W., Gerken G., Giese T., Stallmach A. (2009). TNF-*α* antibody treatment in refractory collagenous sprue: report of a case and review of the literature. *Zeitschrift für Gastroenterologie*.

[14] van Gils T., van de Donk T., Bouma G., van Delft F., Neefjes-Borst E. A., Mulder C. J. J. (2016). The first cases of collagenous sprue successfully treated with thioguanine. *BMJ Open Gastroenterol*.

[15] Riddell J., Hillman L., Chiragakis L., Clarke A. (2007). Collagenous colitis: oral low-dose methotrexate for patients with difficult symptoms: long-term outcomes. *Journal of Gastroenterology and Hepatology*.

[16] Colman R. J., Lawton R. C., Dubinsky M. C., Rubin D. T. (2018). Methotrexate for the treatment of pediatric crohn’s disease: a systematic review and meta-analysis. *Inflammatory Bowel Diseases*.

[17] Ferrara G., Mastrangelo G., Barone P. (2018). Methotrexate in juvenile idiopathic arthritis: advice and recommendations from the MARAJIA expert consensus meeting. *Pediatric Rheumatology*.

